# Influence of Water-Induced Degradation of Polytetrafluoroethylene (PTFE)-Coated Woven Fabrics Mechanical Properties

**DOI:** 10.3390/ma15010001

**Published:** 2021-12-21

**Authors:** Andrzej Ambroziak, Paweł Kłosowski

**Affiliations:** Faculty of Civil and Environmental Engineering, Gdansk University of Technology, 11/12 Gabriela Narutowicza Street, 80-233 Gdansk, Poland; klosow@pg.edu.pl

**Keywords:** construction materials, water-induced degradation, PTFE coated woven fabric, uniaxial tensile test, mechanical properties

## Abstract

The impact of water-induced degradation on the mechanical properties of the chosen two PTFE-coated, glass threads woven fabrics is investigated in this paper. The paper begins with a survey of literature concerning the investigation and determination of coated woven fabric properties. The authors carried out the uniaxial tensile tests with an application of flat and curved grips to establish the proper values of the ultimate tensile strength and the longitudinal stiffness of groups of specimens treated with different moisture conditions. Despite the water resistance of the main materials used for fabrics manufacturing, the change of the mechanical properties caused by the influence of water immersion has been noticed. The reduction in the tensile strength resulting under waterlogged is observed in the range from 5% to 16% depending on the type of investigated coated woven fabric and direction of weft or warp.

## 1. Introduction

**Figure 1:**
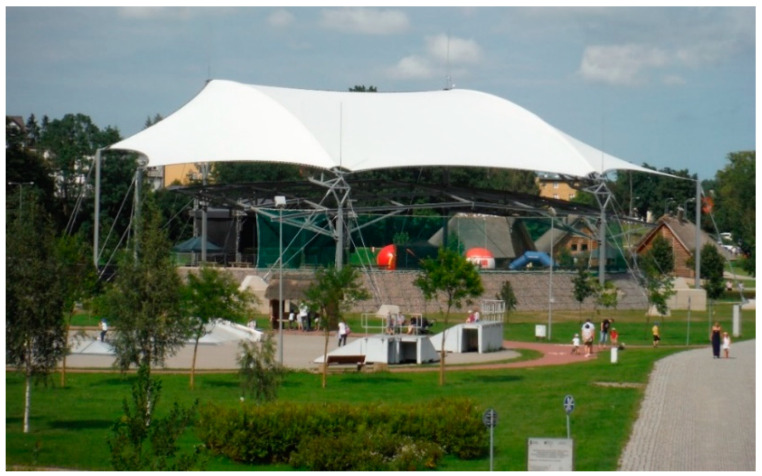
Membrane hanging roof co-designed by the authors in Pruszcz Gdanski, Poland.

The glass threads, polytetrafluoroethylene (PTFE)-coated woven fabrics are one of the most popular types of architectural fabrics applied in membrane structures (see Figure 1) and are increasingly used for facades. The impressive and unordinary membrane structures are designed and built all over the world in different environmental conditions (see e.g., Shopping mall Titan Plaza, Bogotá, Columbia [1], London 2012 Olympic Stadium [2], Winter Garden, Verona, Italy [3]).

The coated woven fabrics called architectural fabrics spark a vital interest in the community of engineers, designers, and scientists to take into consideration, as the subject of different investigations to describe their complex mechanical behavior and influence on thermal and environmental conditions. In the beginning, the authors decided to perform a survey of literature concerning the investigation carried out on coated woven fabrics with topical grouping to show how the widespread research and development area of coated woven fabrics is.

Due to discrete microstructure, non-linear, and inelastic strain-stress relationships of the coated woven fabrics, a large number of the constitutive models are proposed and developed [4]. Hass [5] and Pierce [6] in early works investigated force models of woven fabric in membrane structures. Argyris et al. [7] presented rheological relations and numerical simulations for PVC-coated fabrics. Szostkiewicz-Chatain and Hamelin [8] developed stiffness and inverse identification methods for polyester fabrics. Bridgens and Gosling [9] developed the direct stress-strain material model for coated woven fabrics. Pargana et al. [10] introduced a new constitutive model to deal with coated fabrics. Galliot and Luchsinger [11] performed a shear ramp test for technical fabrics and proposed a non-linear material model. Derewonko et al. [12] proposed a basic material model for numerical simulation based on the uniaxial tensile test for coated fabric material. Ambroziak and Kłosowski [4] proposed the layered dense net model to describe the behavior of coated woven fabrics. Meng and Wu [13] applied a generalized Maxwell model to describe the viscoelastic properties of coated fabrics under relaxation tensile tests. Jekel et al. [14] proposed an inverse bubble inflation test utilizing full displacement field matching to obtain the nonlinear material model. Hegyi et al. [15] proposed a new, two-dimensional phenomenological model for the description of technical woven fabrics. Jekel et al. [16] developed a non-linear orthotropic material model to capture the behavior of PVC-coated polyester under tensile tests. Dib et al. [17] described the non-Newtonian viscous behavior of a coated woven fabric through a proposed material model. Motevalli et al. [18] proposed three hyperelastic orthotropic material models for the simulation of a glass-PTFE membrane. Xu et al. [19] investigated the shear behavior of PTFE-coated fabrics by picture frame tests and proposed the phenomenological model of PTFE-coated fabrics. Zhao et al. [20] proposed a nonlinear constitutive model for the description of the coted woven fabrics under uniaxial tensile loading.

The different types of laboratory tests (e.g., uniaxial and biaxial tensile tests) are performed to a proper description of the behavior of the coated woven fabrics. Reinhardt [21] compared uniaxial and biaxial tensile test results for chosen PVC-coated polyester fabrics. Day [22] performed biaxial tensile tests for coated fabrics at variable stress ratios. Chen et al. [23] carried out biaxial and uniaxial tensile tests for polyester, PVC-coated woven fabrics. Zouari et al. [24] described the behavior of architectural fabrics under biased tensile tests. El-Messiry and Youssef [25] investigated the relation of the strain–stress contours distribution in a tensioned fabric under a central force. Derewońko et al. [26] investigated the effect of the width of a specimen cut out from coated fabric subjected to uniaxial tensile on the transverse deformation of the material. Ambroziak and Kłosowski [27] performed biaxial and uniaxial tests for AF9032 architectural fabric. Chen et al. [28] experimented on a Uretek321A envelope fabric under biaxial and uniaxial tensile tests. Chen et al. [29] determined for Uretek3216 fabric the Poisson’s ratio change under biaxial tests. Bögner-Balz et al. [30] described materials used in textile architecture and showed the mechanical behavior of coated fabrics. Ambroziak [31] investigated mechanical properties of Precontraint 1202 polyester coated fabrics under biaxial tensile tests with different load ratios. Ambroziak [32] performed the biaxial and uniaxial experimental tests for FR 8540 polyester type fabric. Chen and Chen [33] predicted the uniaxial tearing strength for Uretek3216LV coated fabrics. Wu et al. [34] focused on the uniaxial and biaxial mechanical properties for ethylene tetrafluoroethylene foil and suggested optimal partial safety factors for this material. Shi et al. [35] proposed the novel test method of the biaxial bias-extension method and studied the shear behavior of chosen architectural coated fabrics. Chen et al. [36] carried out tensile tests for coated biaxial warp-knitted fabric with different bias angles to estimate the Poisson’s ratios. Shi et al. [37] focused on the biaxial properties of PTFE coated architectural fabric membrane with a new specimen and digital image correlation method. Chen et al. [38] examined the warp-knitted fabric PVDF subjected to biaxial loads and described mechanical behaviors under multiple stress ratios. Gao et al. [39] proposed a stress-ratio dependence coefficient to represent the proportion of elongation and shrinkage in different stress ratios on a biaxial monotonic tensile test of PVDF-coated fabrics.

Besides uniaxial and biaxial tensile tests a cyclic, creep, tearing, etc. tests are carried out to show and measure additional complex behaviors of coated woven fabrics. Ansell et al. [40] investigated PTFE-coated woven fabrics exposed to cyclic environmental and creep stresses aging in artificial and natural environments. Ambroziak and Kłosowski [41] studied the mechanical properties of polyvinyl chloride-coated fabric under cyclic tests. Zhang et al. [42] focused on the tensile properties of coated fabrics under monotonous forces and cyclic loads. Wang et al. [43] carried out the cycling-uniaxial tensile tests based on the mono-uniaxial tensile experiments. Junhao et al. [44] investigated off-axial tensile behaviors of polytetrafluoroethylene-coated woven glass fibers under different loading rates. Żerdzicki et al. [45] presented the mechanical response of the VALMEX fabric during the cyclic loading-unloading and reloading experiments. Zhao et al. [46] proposed a method for long-term uniaxial creep tests of membrane materials. Uhlemann et al. [47] developed a saturation analysis procedure to assess the mechanical saturation behavior of five biaxial 1000-load cycle saturation of PES-PVC architectural fabrics. Eun et al. [48] investigated the mechanical properties of PVC-coated fabrics with various viscosity PVC resins and studied the mechanical properties of PVC-coated fabrics with various weaving structures such as plain weave structure, matt weave structure. Chen et al. [49] described uniaxial tearing tests, the influence of slit parameters, off-axis angles, and loading speeds on tearing behavior and strength. Elthan [50] investigated structural parameters affecting the tear strength of the cotton woven and duct fabrics and PVC coated fabrics. Chen et al. [51] investigated the fracture failure analysis on plain-woven laminated fabrics used in stratospheric airship structures. Sun et al. [52] described the tearing residual strength models of cracked PVC coated fabric and PTFE coated fabric under uniaxial tensile tests. He et al. [53] investigated biaxial tearing properties of PVC-coated fabrics using digital image correlation. Sun et al. [54] experimental and analytical investigation on central crack tearing properties of PTFE coated fabric. Xu et al. [55] described the puncture resistance behaviors of architectural coated fabric and its influence on the effects of boundary condition, penetrator shape, yarns angle, puncture rate, the weight percentage of fiber, and coating on puncture resistance behaviors of coated fabric. Li et al. [56] described the central tearing behaviors of PVDF coated fabrics with initial notches under the influence of notches number, notches length, and notches orientation.

Generally, the final product used directly in the tensile structures is tested. Nevertheless, some works investigated also uncoated fabric. Chen et al. [57] investigated coated fabrics before and after coating under tensile tests and compared their mechanical properties. Wang and Zhang [58] compared the tensile strength of uncoated and coated technical fabrics. Wang et al. [59] focused on the stab failure behavior of coated and uncoated woven fabric. These scientific works allow evaluating the influence of the coating application on the behavior of coated fabrics.

Investigations on a description of material and mechanical properties for different coated woven fabrics are widely conducted also. Bassett et al. [60] reviewed various experimental approaches for coated fabrics to assess the mechanical parameters. Kłosowski et al. [61] determined the viscoplastic properties of coated fabrics. Kłosowski et al. [62] applied a nonlinear viscoelastic model for the PVC-coated polyester fabric. Żerdzicki et al. [63] applied the elasto-viscoplastic Bodner-Partom model for Valmex coated fabric. Uhlemann et al. [64] described and compared two different methods of determining stiffness parameters for three types of PVC-coated fabrics under biaxial tensile tests. Chen et al. [65] investigated the penetration-resistant capability of PVC-coated polyester fabric using a pneumatic cannon system. Van Craenenbroeck et al. [66] described the material properties of PVC-coated polyester fabric and their validation. Meng et al. [67] applied the Monte Carlo simulation algorithm based on the Ising model to analyze the force-displacement curve with different regions under uniaxial tensile tests. Meng et al. [68] applied the simplified maximum stress criterion and a modified Tsai-Hill criterion to predict the failure of the coated fabrics under on-axial and off-axial tension. Zhao et al. [69] described an in situ method to determine the stress distribution of an inflatable membrane structure using a force-finding method based on measured configuration. Dinh et al. [70] proposed a method to combine different stages in the design and analysis of membrane structures at tension within the shape optimization framework. Kłosowski et al. [71] analyzed technical fabrics’ behavior thoroughly through viscoplastic Bodner-Partom constitutive law. Chen et al. [72] examined coated URETEK 3216 LV fabrics under mono-uniaxial, biaxial, and uniaxial cyclic loads. Ambroziak and Kłosowski [73] identified mechanical properties for the sail technical woven fabric style 480 AP (yacht sailcloth polyester) with a medium tempered optimized finish. Xu et al. [74] investigated off-axis mechanical behaviors of PVC-coated fabrics by digital image correlation technology to measure the shape, displacement, and strain. Chen et al. [75] developed a MATLAB program for achieving complex deformation properties and accurate elastic parameters of woven fabrics under biaxial tests. Khaothong [76] used the Taguchi method for investigated factors affecting the strength of welding seam through hot air for PVC-acrylic coated polyester fabrics.

A large number of investigations are also performed on assessment changes of coated woven fabric properties influences by temperature, aging processes, or other factors. Zhang et al. [77] determined mechanical properties and applied a temperature reduction factor for coated woven fabrics. Ambroziak and Kłosowski [78] identify mechanical and thermal properties of PVC-coated polyester fabrics in the range of temperature form −30 °C to +70 °C. Jabbari et al. [79] described the thermal degradation, effect of aerogel-content, thermal insulating properties, surface characteristics, and tensile properties of polyester fabrics. Zhang et al. [80] performed research on the stress-relaxation behaviors of PTFE coated fabrics under temperatures from 23 °C to 70 °C. Meng et al. [81] examined thermal distribution and deformation of coated woven fabrics. Yu et al. [82] studied the mechanical properties of polyvinyl chloride PVC-coated woven fabric exposure to temperature ranges from 20 °C to 170 °C under the uniaxial tensile test. Sun et al. [83] investigated the temperature effects (ranging from 23 °C to 250 °C) on the mechanical properties of the polyester-coated fabric membrane materials under uniaxial loading. Li et al. [84] investigated the effects of natural aging on the properties of a polyvinylidene fluoride PVDF-coated fabric. Zhang and Zhang [85] described the degradation behaviors of PVDF-coated polyesters by artificial accelerated tests and studied the effects of environmental factors and loading conditions. Zerdzicki et al. [86] investigated the influence of service aging on polyester-reinforced polyvinyl chloride-coated fabrics under uniaxial tensile, biaxial tensile, and long-term creep tests. João et al. [87] studied the durability of two architectural membranes at the initial stages of environmental exposure, one with polyester fiber coated with polyvinylchloride and the other with glass fiber coated with PTFE. Yang et al. [88] performed statistical characteristics of naturally aged polyvinylidene fluoride PVDF-coated fabrics’ mechanical properties and described reliability indexes based on the central point method and Rackwitz-Fiessler method. Klosowski et al. [89] studied thermal aging evaluation for polyester-reinforced and polyvinyl chloride coated fabrics and specified the mechanical properties of the aged fabric using the identified linear piecewise model and by the Bodner–Partom model. Dobilaitė et al. [90] performed an experimental study of the tearing behavior of PVC-coated fabrics exposed to different accelerated aging conditions. Shang et al. [91] examined three groups of PVDF-coated fabrics removed from different membrane structures which were used for 15, 16, and 19 years. Toyoda et al. [92] pointed, that a decrease of the tensile strength of PTFE coated glass woven fabric is observed when fabric was immersed into hot water. Razak [93] evaluated the weather ability of PTFE- and PVC-coated fabrics under tropical climate on outdoor exposure tests for 2 years. Asadi et al. [94] investigated water diffusion and water-induced degradation mechanisms (on the tensile strength and the breaking strain) of glass/polytetrafluoroethylene (PTFE) architectural fabrics.

The literature concerning coated woven fabrics is very extensive, nevertheless, the authors pay attention to chosen studies concerning the wide and comprehensive studies on the coated woven fabrics. The literature review indicates research concerning constitutive models, different types of laboratory tests, determination of material and mechanical properties, assessment of coated woven fabric mechanical properties changes under different factors, etc. The studies concerning water-induced degradation on coated woven fabrics are limited. Therefore, there is a necessity for carrying out detailed and extended research in this area. Continuous development in textile and membrane materials provides an impulse to conduct new investigations on coated woven fabrics. It can be mentioned that coated fabrics are still developed, tested, and investigated by engineers, researchers, and scientists.

The fabrics producers usually provide the designer with strength parameters for dry material. But when the final construction is exposed to changing weather conditions, especially during the rain or winter season, when rain or wet snow can remain for a long time on the roof surface, the tensile strength can be reduced. There are many factors, which can affect the mechanical properties and final behavior of the coated woven fabrics. Environmental conditions that occur during coated woven fabric exploitation (e.g., build-in membrane or hanging structures) seem to be a prominent factor. One of that factors is rain and high moisture. The present study is aimed at the determination of the influence of water-induced degradation on mechanical parameters of chosen PTFE-coated woven fabrics on changing of mechanical properties under uniaxial tensile tests with an application of two types of grips (flat and curved). This paper provides scientists, engineers, and designers with experimental assessments of the influence of water-induced degradation on mechanical parameters (tensile strength) of investigated two types of PTFE-coated woven fabrics.

## 2. Materials and Methods

Two woven fabrics having glass threads coated by PTFE were chosen for laboratory tests. According to manufacturers’ (Saint-Gobain Performance Plastics, Merrimack, United States and Verseidag-Indutex GmbH, Krefeld, Germany) declarations given in Table 1, their basic properties are similar. Both architectural fabrics have similar construction and total weight (difference about 0.7%) with about 7% difference in weight of base threads net per unit area. As declared by manufacturers tensile strength in the warp and weft directions differs about 5% and 10% between S-type and B-type. In the year 2006, both PTFE-coated woven fabrics were considered as the potential construction material for the membrane roof of the Forest Opera in Sopot (Poland).

**Figure 2:**
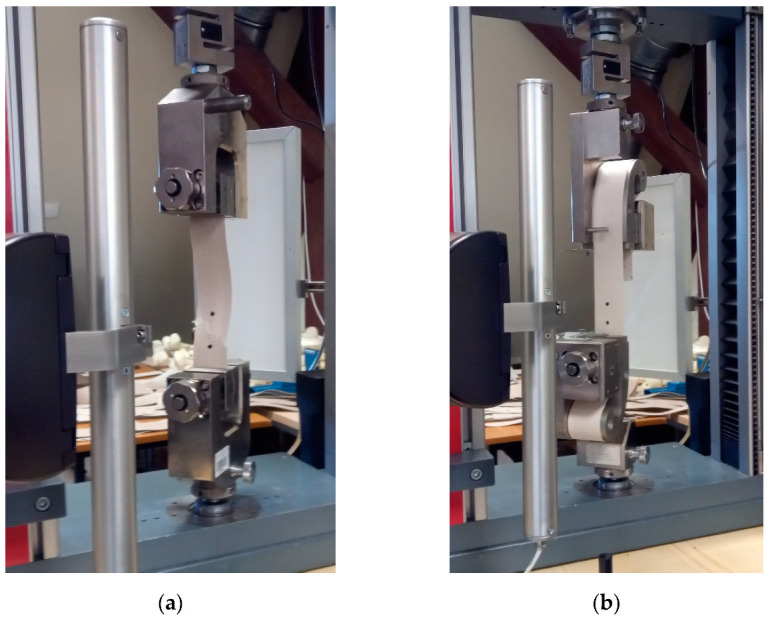
Uniaxial laboratory tests stand. Coated fabric samples fixed: (**a**) in flat grips; (**b**) in curved grips.

The uniaxial tensile tests were conducted on the Zwick 020 mechanical testing machine (Zwick Roell GmbH, Ulm, Germany), under the video extensometer control based on the digital image correlation method with the base of the optical extensometer of about 50 mm. The results from the video extensometer were used to identify the fabric stiffness functions. The specimens had 50 ± 1 mm width, and the active length equal to 200 ± 1 mm for application of flat grips (see Figure 2a), and the total length of 900 ± 1 mm for curved grips (see Figure 2b), respectively. The tests have been performed according to guidelines in the ISO 1421:2016 standard [95] for the strip method, with the displacement rate of the upper grip of 100 mm/min (see Figure 2a). Each type of test has been repeated at least three times.

Three main groups of coated fabric specimens were tested. All specimens were cut out from the bales of fabric stored for a long time in the laboratory in room conditions. For the first group, the fabric specimens were cut in the warp and weft direction from the base material (virgin fabric). Then they were, measured, weighed, and then immediately tested. Results for this group are denoted S or B regarding S type or B type coated fabrics. The two remaining groups of specimens fabric were immersed into room temperature water for two weeks. The immersion period was twice longer than taken by Asadi et al. [94]. After two weeks they were taken out from the water. Their surface was dried using absorbent paper. The specimens of the second group were weighted and then immediately tested. This group is denoted as S_wet or B_wet reference to coated fabrics S or B types tested as wet. The third group of fabric specimens, after two weeks of water soaking, was left in the room conditions to dry out for the following one week and then weighted and tested. The last group is denoted as S_air-dried and B_air-dried concerning S type or B type coated fabrics subjected to waterlogging and process of air-drying.

To recognize the importance of mechanical parameters changes according to moisture conditions and history of water impact on the coated fabric, the one-way ANOVA statistical analysis (see e.g., [96,97]) was performed. An ANOVA test is a way to find out whether the results of laboratory tests obtained for defined groups of specimens differ significantly from each other. The tests were divided into three groups dry (base specimens), wet, and air-dried, and mechanical properties of each group were investigated. To perform ANOVA tests it is necessary to check whether data have the normal distribution and whether the variances of data sets have the same value. Therefore, before the ANOVA test, the assumptions of the normal distribution of results (Shapiro-Wilk test, see e.g., [98]) and the equal variance test (Brown-Forsythe test, see e.g., [99]) were checked. If the data sets would not pass the Shapiro-Wilk test and the Brown-Forsythe test, the ANOVA analysis could not be performed. In all presented results both mentioned tests were passed. The ANOVA test was always performed with the parameter of power performance *α* = 0.05.

## 3. Laboratory Test Results and Discussion

### 3.1. Measurements of Surface Weight

After weighing (with an accuracy of ±0.01 g) and dimension measuring (with an accuracy of ±1 mm) for each sample dry, after soaking, and after air-drying were determined the total mass per unit area for chosen types of PTFE coated woven fabrics. The results of measurements of surface weight determining are collected in Table 2. The mean value of the mass per unit area as well as the standard error of the mean of the specified range is presented. The measured total mass of the dry coated fabric specimens was about 2% lower for S-type and 5% higher for B-type fabric than declared by manufacturers. The determined total mass per unit area for the fabric specimens subjected to 2-weeks soaking process in water was about 1.4 ÷ 2% higher than its initial weight. The dried specimens have the total mass per unit area about 0.5 ÷ 0.8% lower than the initial what can mean that moisture of the rolled fabric (in that form the fabrics have been stored) was a little bit higher than the dried specimens (higher values were obtained for the B-type fabric). It can be concluded that the lowest total mass per unit area for air-dried fabric specimens than for dry fabric specimens may be connected with the fact that rolled fabric cut into smaller pieces gives off the imprison moisture by cross-section face fastest than by surfaces when are rolled and stored. Additionally, a deviation in the total mass per unit area of the coated fabric itself (e.g., a slight difference in coating thickness) influences the final weight of the coated fabrics.

The comparison of the surface weight of investigated groups of fabrics showed the statistically important differences for all three groups for the B type of fabric *p* < 0.05 and not the important difference between dry and air-dried groups for the S type (*p* = 0.218). That means that the S type of fabric has almost the same density before soaking and after the air-drying process from the statistical point of view.

### 3.2. Uniaxial Tensile Tests

**Figure 3:**
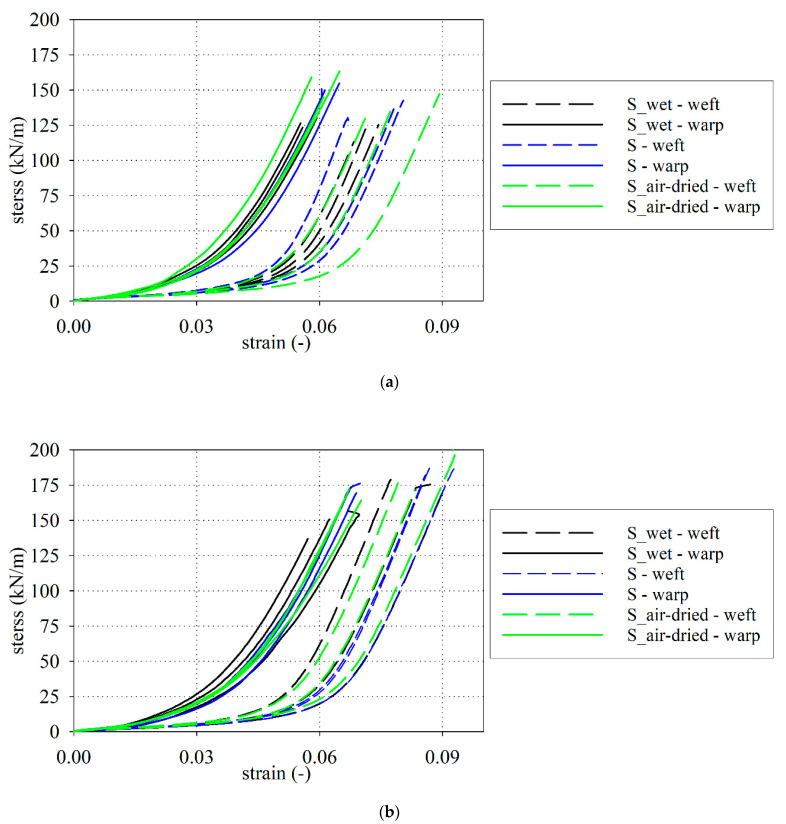
Stress-strain curves, S type fabric: (**a**) flat grips; (**b**) curved grips.

**Figure 4:**
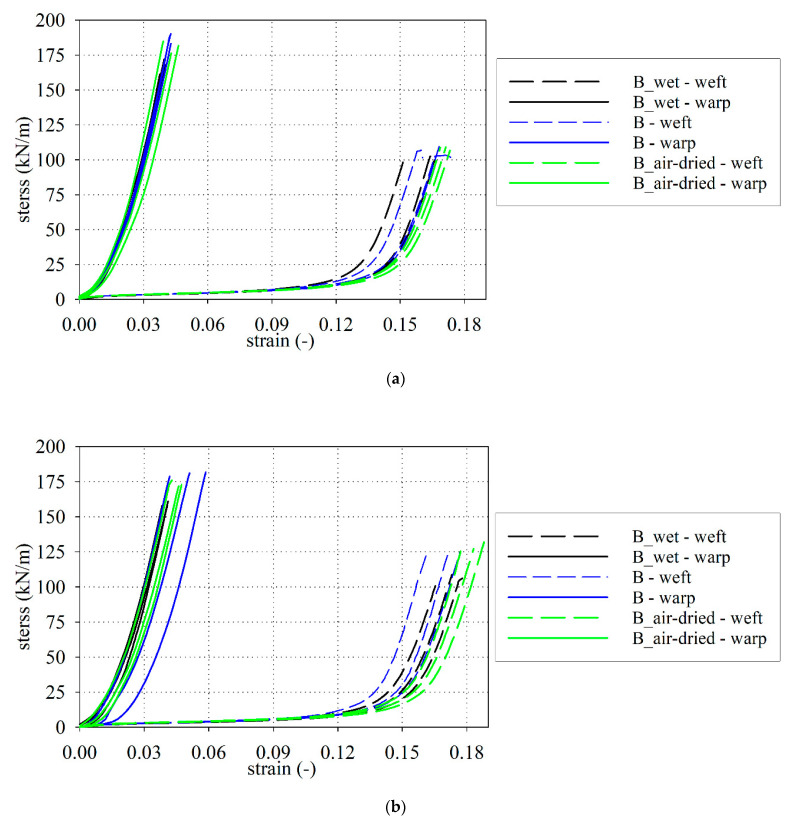
Stress-strain curves, B type fabric: (**a**) flat grips; (**b**) curved grips.

The uniaxial tensile tests were performed to evaluate the tensile strength and the related elongation at break, as well as the fabric stiffness in the specified ranges of deformation. The results (time, displacements measured by extensometer, and the applied force) have been recorded by a computer and recalculated to the stress-strain functions presented in Figure 3 for the S type fabric and in Figure 4 for the B type fabric. Figure 3a and Figure 4a are given results for S type and B type fabric with the application of flat grips, respectively. The results for curved grips are shown in Figure 3b and Figure 4b.

### 3.3. Elongation at Break and Tensile Strength

**Figure 5:**
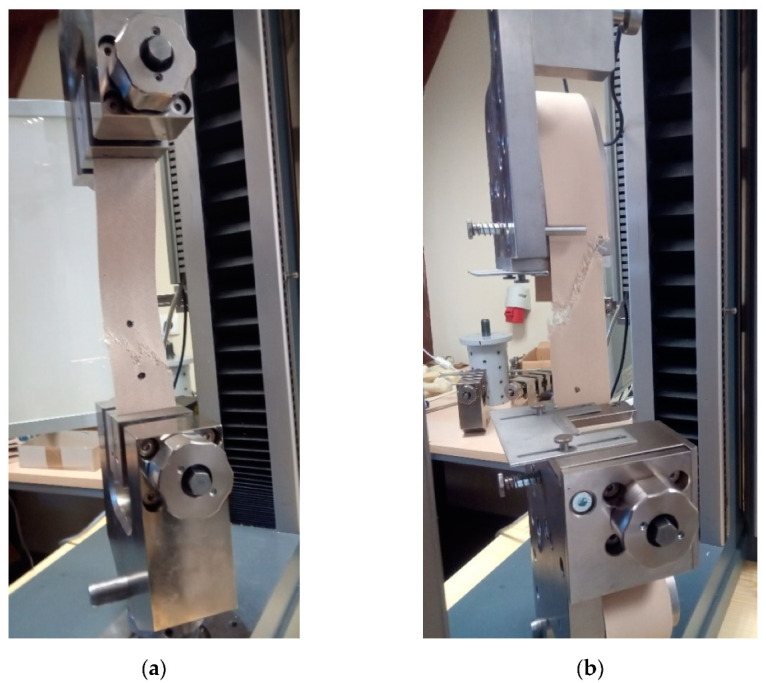
The typical form of specimens failure under uniaxial tensile tests: (**a**) flat grips; (**b**) curved grips.

Based on the performed uniaxial tensile tests the elongation at break and the tensile strength are specified for fabrics investigated under different conditions: dry, wet, and air-dray. For evaluation of the tensile strength, the type of used grips may play an important role. The ISO 1421 standard [95] does not specify what kind of grips should be used. It is required to avoid close to jaw breaks only, see Figure 5 where permitted forms of failure are shown. Nevertheless, the tensile strength obtained using flat grips is usually lower than for curved grips due to close grips stress concentrations of fabric specimens and appearing breaks of fabric specimens close to flat grips during tests. Therefore to compare the moisture influence both sets of results are separated. The uniaxial tensile test results for three groups of fabric specimens (base: S and B, wet: S_wet and B_wet, and air-dried: S_ air-dried and B_ air-dried) are presented in Appendix A Table A1, Table A2 and Table A3 for S type fabric and in Table A4, Table A5 and Table A6 for B type fabric.

The large differences between tensile strength (see e.g., in weft direction between flat and curved grips for S, S_wet, S_air-dried, B, B_air-dried) are connected with exhibited of breaks of fabrics specimens near the grips thus the flat grips results sometimes cannot be treated as proper value of the tensile strength of coated fabrics. Nevertheless, in this investigation, for the flat grips results for the B-type fabric in the warp direction, the tensile strength is greater than for curved grips.

**Figure 6:**
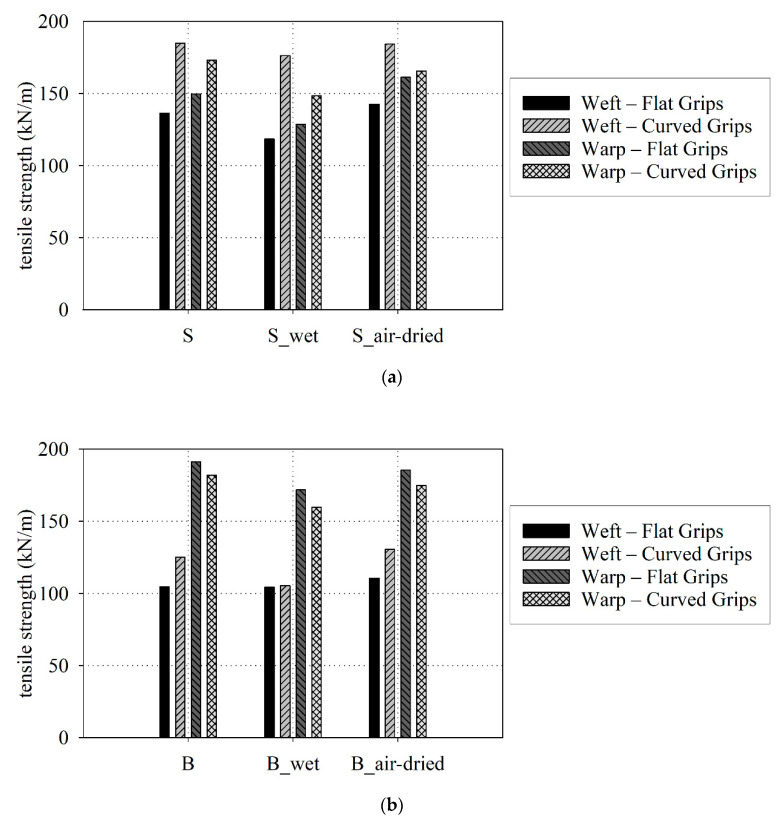
Tensile strength: (**a**) S type fabric; (**b**) B type fabric.

The specified mean values of tensile strength of the dry S-type fabric (base specimens) for weft and warp directions is 185 ± 2 kN/m and 173.1 ± 2 kN/m (see Table A1) and they are higher by 20% and 2% than the values declared in Table 1. For the dry B-type fabric (base specimens) the determining tensile strength is 11% lower for the weft direction and 20% higher for warp direction (see Table A4) than declared by the manufacturer, see Table 1. The mean values of the tensile strength for the wet fabrics (S_wet and B_wet) are reduced, see Figure 6a,b. In the case of the S type fabric is reduced by 5% (to 176.4 ± 2 kN/m) in the weft direction, and 20% (to 148.6 ± 6 kN/m) for the warp direction, see Table A2. The same effect has been obtained for the B-type fabric—reduction by 16% (to 105.5 ± 3 kN/m) in the weft direction and reduction by 11% (to 171.8 ± 2 kN/m) for the warp direction, see Table A5. The air-dried fabric specimens obtained a higher mean value of tensile strength than wet fabric specimens, see Figure 6a,b. The difference in mean tensile strength for air-dried fabrics is about 4% compared to base fabric specimens, see Table A3 and Table A6.

The importance of the influence of water-induced degradation mechanisms on the tensile strength has been verified by the ANOVA one-way statistical analysis. The change of the tensile strength should be analyzed for curved grips as an application of flat grips usually generates the jaws breaks and cause a reduction of the tensile strength, (see Figure 5). For the S-type of fabric more important influence of moisture can be observed in the weft direction, where the important differences have been noticed between dry/air-dried and wet/dry groups *p* < 0.05 (for dry/wet *p* = 0.377). For warp direction the important difference has been obtained for dry/wet comparison only (air-dried/wet *p* = 0.054, dry/air dried *p* = 0.248). For the B-type of fabric, the relevant differences have been obtained for all compared groups.

### 3.4. Determination of the Longitudinal Stiffnesses

**Figure 7:**
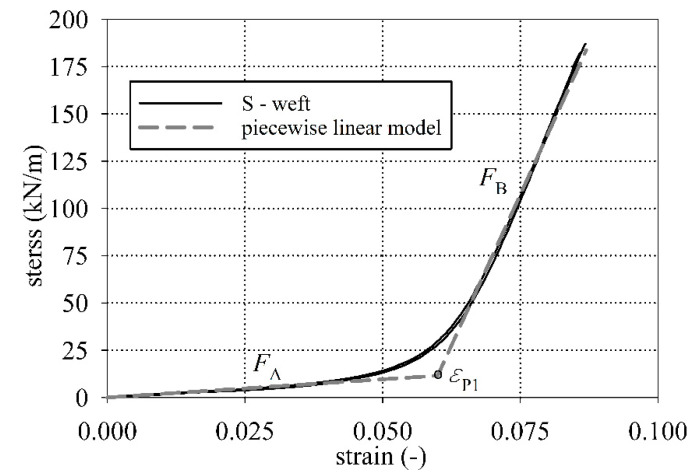
Graphical concept of the piecewise linear model identification.

Analyzing the stress-strain curves for the S and B types of PTFE-coated fabrics (see Figure 3 and Figure 4), the characteristic points of the curvature change can be observed. To describe the stress-strain curves, it is possible to use the piecewise linear model (see e.g., [41]). In this concept, it is necessary to specify the longitudinal stiffnesses *F_i_* (kN/m) and the intersection points $\varepsilon_{Pi}$ (-) which define the range of applicability of the certain longitudinal modulus, see Figure 7. The stiffness coefficients *F_i_* (kN/m) were determined from the linear approximation of the stress-strain relationship using the piecewise linear model.

**Figure 8:**
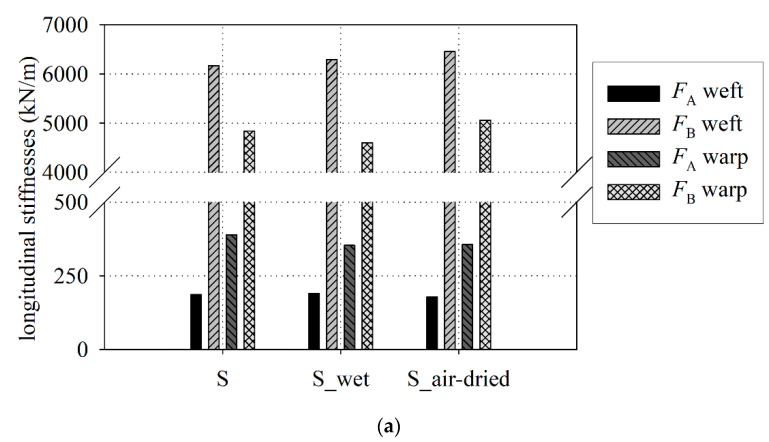

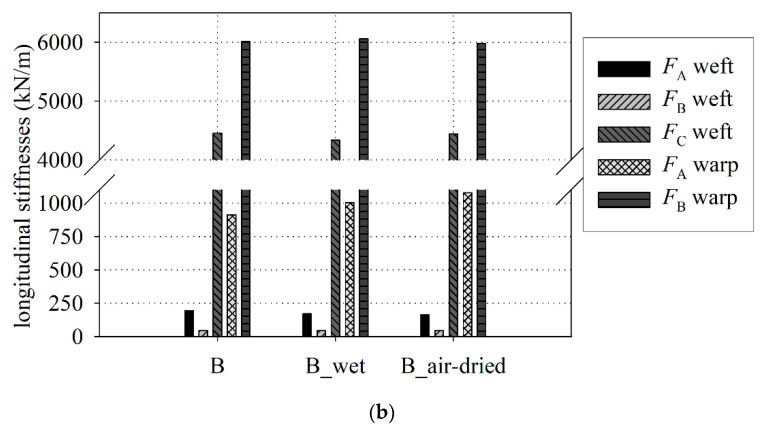
Longitudinal stiffnesses: (**a**) S type fabric; (**b**) B type fabric.

The application of the video extensometer made it possible to unify the results of tests for flat and curved grips into one group. The video extensometer measures strain in the middle part of the specimen without a touch of the specimen, far from the grips, see Figure 2b. Therefore obtained strain values used for stiffness identification are determined more precisely. Therefore, the comparison of the waterlogged influence of the fabric stiffness is assumed as not kind of grips dependent. The longitudinal stiffnesses were determined and presented in Figure 8a,b for S type and B type fabrics, respectively. The tabular results are collected in Table A7 for S-type fabric and in Table A8 for B-type fabric, respectively.

Taking into account the mean stiffnesses of *F*_B_ and *F*_C_ it may be concluded that the difference of stiffnesses between the dry and wet fabrics ranges from 2% (for weft) to 5% (for warp) for the S-type fabric, see Table A7. The difference for the B-type fabric ranges maximally from 0.8% (for warp) to 3% (for weft), see Table A8. The mean stiffness *F*_A_ of the wet S-type fabric is vary by about 2% for weft and 9% for warp in comparison with dry properties, while for the wet B-type fabric differs by about 10% (for weft and warp), see Figure 8. The influence of water on the investigated PTFE-coated fabric stiffness is less essential than for the tensile strength. According to performed ANOVA analysis, it can be stated that the differences in the mean values among the investigated groups (dry, wet, and air-dry) are not great enough to exclude the possibility that the difference is due to random sampling variability; there is not a statistically significant difference.

## 4. Conclusions

In the present paper, two chosen architectural fabrics constructed from glass threads and have the PTFE coating (with some additional thin layers) are investigated. The main materials used to produce the fabrics are water-resistant. Nevertheless, the final product is sensitive to the level of moisture. Based on performed investigation the following conclusions may be drawn:

- The reduction in the tensile strength resulting under waterlogged was observed in the range from 5% (for weft) to 14% (for warp) for S-type fabric and from 16% (for weft) to 10% (for warp) for B-type fabric, respectively. A similar conclusion came from the authors of [94], where the change in the tensile strength under influence of water was specified nearby 20%.
- The mechanical properties of investigated PTFE coated fabric under water-induced are degraded not only via in-plane watering at unsealed cut edges but also via out-of-plane watering through the coating.
- The influence of moisture is not the same for different physical parameters. The most sensitive is the tensile strength.
- Even small moisture changes as it has been reported between the base fabric and air-dried material cause statistically important changes in the tensile strength. The changes in fabric stiffness are much less sensitive and from the statistical point of view, they can be neglected.
- Changes in the stress-strain curve characteristic under different moisture conditions of PTFE coated fabrics cannot be indicated due to the significant scatter of obtained results, see Figure 3 and Figure 4.
- The ANOVA analysis indicated that the longitudinal stiffnesses differences among the investigated groups (dry, wet, and air-dry) are not statistically significant. Future investigations should take into account a more large number of specimens to confirm differences.
- Types of grips used in tensile tests influenced the determined values of the tensile strength. The type of grips is not important during the stiffness of fabric determination. Determination of the tensile strength should be rather performed by using the curved grips to avoid the close-to-grip breaks in coated fabric specimens that are often observed when the flat grips are used. Such close-to-grip breaks reduce the tensile strength value.

The paper provides scientists, civil engineers, and designers with knowledge of water-induced degradation of chosen PTFE-coated woven fabrics’ mechanical properties and requirements to applying curved grips during the process of determination of tensile strengths. Determination of the proper values of tensile strengths of coated woven fabrics under environmental conditions requires assessment of the dry and wet conditions. The coated woven fabrics producers should provide the designer with strength parameters for dry and wet material. The obtained results encourage the authors to continue the research directed towards understanding mechanisms of water-induced degradation of coated woven fabrics’ mechanical properties, based on an extension of uniaxial tensile tests and biaxial tensile tests for waterlogged fabrics.

## Figures and Tables

**Table 1:** Properties of investigated PTFE coated woven fabrics.

Properties	S Type	B Type
Total Mass per Unit Area (g/m^2^)	1540	1550
Tensile Strength (kN/m): Warp	170	160
Tensile Strength (kN/m): Weft	156	140
Base Coat	PTFE	PTFE
Weight per Unit Area of Base Fabric (g/m^2^)	625	670
Yarn Count (yarn/cm): Warp	7.1	8
Yarn Count (yarn/cm): Weft	7.5	7.5
Translucency at 550 nm (%)	9	8–11

**Table 2:** Total mass per unit area (g/m^2^).

	Dry (Base Specimens)	Wet	Air-Dried
S-type	1511 ± 4	1532 ± 4	1503 ± 5
B-type	1638 ± 4	1670 ± 4	1625 ± 4

## Data Availability

All laboratory test results are presented in Figure 3 and Figure 4. On the request, the numerical version of the results will be provided.

## Appendix A

Results of elongation at break and tensile strength for the base, wet, and air-dried specimens of the A-type and B-type coated fabrics are presented in Table A1, Table A2, Table A3, Table A4, Table A5 and Table A6. The determined longitudinal stiffnesses of S type and B type fabrics are collected in Table A7 and Table A8, respectively.

**Table A1:** Elongation at break and tensile strength—base specimens S-type fabric.

Specimens No.	Weft—Flat Grips SWp		Weft—Curved Grips SW		Warp—Flat Grips SOp		Warp—Curved Grips SO	
	Rupture Strain	Tensile Strength	Rupture Strain	Tensile Strength	Rupture Strain	Tensile Strength	Rupture Strain	Tensile Strength
	*-*	kN/m	*-*	kN/m	*-*	kN/m	*-*	kN/m
1	0.0804	142.5	0.0859	182.2	0.0622	147.7	0.0694	171.0
2	0.0670	128.7	0.0929	187.0	0.0689	156.2	0.0745	171.3
3	0.0784	138.0	0.0868	187.1	0.0567	145.6	0.0723	177.2
mean	0.0753 ± 0.0042	136.4 ± 4	0.0885 ± 0.0022	185 ± 2	0.0626 ± 0.0035	149.8 ± 3	0.0721 ± 0.0015	173.1 ± 2

**Table A2:** Elongation at break and tensile strength—wet S type fabric.

Specimens No.	Weft—Flat Grips SWp_Wet		Weft—Curved Grips SW_Wet		Warp—Flat Grips SOp_Wet		Warp—Curved Grips SO_Wet	
	Rupture Strain	Tensile Strength	Rupture Strain	Tensile Strength	Rupture Strain	Tensile Strength	Rupture Strain	Tensile Strength
	*-*	kN/m	*-*	kN/m	*-*	kN/m	*-*	kN/m
1	0.0738	112.4	0.0939	175.1	0.0508	129.6	0.0573	138.0
2	0.0905	122.1	0.0776	180.3	0.0485	122.4	0.0625	151.1
3	0.0989	120.8	0.1253	173.8	0.0448	134.1	0.0700	156.9
mean	0.0878 ± 0.0074	118.5 ± 3	0.0989 ± 0.0140	176.4 ± 2	0.0480 ± 0.0018	128.7 ± 3	0.0633 ± 0.0037	148.6 ± 6

**Table A3:** Elongation at break and tensile strength—air-dried S type fabric.

Specimens No.	Weft—Flat Grips SWp_Air-Dried		Weft—Curved Grips SW_Air-Dried		Warp—Flat Grips SOp_Air-Dried		Warp—Curved Grips SO_Air-Dried	
	Rupture Strain	Tensile Strength	Rupture Strain	Tensile Strength	Rupture Strain	Tensile Strength	Rupture Strain	Tensile Strength
	*-*	kN/m	*-*	kN/m	*-*	kN/m	*-*	kN/m
1	0.0896	149.4	0.0800	177.0	0.0659	169.8	0.0705	166.1
2	0.0718	134.7	0.1290	202.5	0.0619	150.8	0.0651	158.5
3	0.0782	143.7	0.0835	173.5	0.0584	163.6	0.0913	172.4
mean	0.0799 ± 0.0052	142.6 ± 4	0.0975 ± 0.0158	184.3 ± 9	0.0480 ± 0.0018	161.4 ± 6	0.0757 ± 0.0079	165.7 ± 4

**Table A4:** Elongation at break and tensile strength—base specimens B type fabric.

Specimens No.	Weft—Flat Grips BWp		Weft—Curved Grips BW		Warp—Flat Grips BOp		Warp—Curved Grips BO	
	Rupture Strain	Tensile Strength	Rupture Strain	Tensile Strength	Rupture Strain	Tensile Strength	Rupture Strain	Tensile Strength
	*-*	kN/m	*-*	kN/m	*-*	kN/m	*-*	kN/m
1	0.1736	101.8	0.1761	126.3	0.0436	189.0	0.0592	184.1
2	0.1684	111.0	0.1755	123.4	0.0432	192.4	0.0401	179.7
3	0.1606	101.4	0.1768	125.6	0.0387	192.5	0.0512	181.7
mean	0.1675 ± 0.0038	104.7 ± 3	0.1761 ± 0.0007	125.1 ± 1	0.0418 ± 0.0016	191.3 ± 1	0.0502 ± 0.0055	181.9 ± 1

**Table A5:** Elongation at break and tensile strength—wet B-type fabric.

Specimens No.	Weft—Flat Grips BWp_Wet		Weft—Curved Grips BW_Wet		Warp—Flat Grips BOp_Wet		Warp—Curved Grips BO_Wet	
	Rupture Strain	Tensile Strength	Rupture Strain	Tensile Strength	Rupture Strain	Tensile Strength	Rupture Strain	Tensile Strength
	*-*	kN/m	*-*	kN/m	*-*	kN/m	*-*	kN/m
1	0.1673	107.8	0.1731	108.7	0.0428	174.7	0.0759	159.0
2	0.2261	104.6	0.1860	106.1	0.0388	168.3	0.0357	158.0
3	0.1570	101.2	0.1655	101.9	0.0403	172.3	0.0417	162.8
mean	0.1835 ± 0.0215	104.5 ± 2	0.1749 ± 0.0104	105.5 ± 3	0.0407 ± 0.0011	171.8 ± 2	0.0511 ± 0.0125	159.9 ± 1

**Table A6:** Elongation at break and tensile strength—air-dried B type fabric.

Specimens No.	Weft—Flat Grips BWp_Air-Dried		Weft—Curved Grips BW_Air-Dried		Warp—Flat Grips BOp_Air-Dried		Warp—Curved Grips BO_Air-Dried	
	Rupture Strain	Tensile Strength	Rupture Strain	Tensile Strength	Rupture Strain	Tensile Strength	Rupture Strain	Tensile Strength
	*-*	kN/m	*-*	kN/m	*-*	kN/m	*-*	kN/m
1	0.1702	111.9	0.1834	133.4	0.0430	183.2	0.0477	174.7
2	0.1736	108.1	0.1834	128.5	0.0485	185.8	0.0429	176.0
3	0.1745	112.0	0.1780	130.3	0.0397	187.4	0.0463	173.6
mean	0.1728 ± 0.0013	110.7 ± 1	0.1816 ± 0.0018	130.7 ± 1	0.0437 ± 0.0026	185.5 ± 1	0.0456 ± 0.0014	174.8 ± 1

**Table A7:** Longitudinal stiffnesses of S type fabric.

	S		S_Wet		S_Air-Dried	
	Weft	Warp	Weft	Warp	Weft	Warp
	(kN/m) (-)	(kN/m) (-)	(kN/m) (-)	(kN/m) (-)	(kN/m) (-)	(kN/m) (-)
*F*_A_ $\varepsilon_{P1}$	187 ± 5 0.059 ± 0.002	390 ± 12 0.035 ± 0.001	191 ± 5 0.056 ± 0.002	354 ± 12 0.035 ± 0.002	179 ± 5 0.059 ± 0.002	357 ± 4 0.037 ± 0.001
*F*_B_	6168 ± 63	4842 ± 137	6294 ± 102	4603 ± 92	6454 ± 101	5064 ± 60

**Table A8:** Longitudinal stiffnesses of B type fabric.

	B		B_Wet		B_Air-Dried	
	Weft	Warp	Weft	Warp	Weft	Warp
	(kN/m) (-)	(kN/m) (-)	(kN/m) (-)	(kN/m) (-)	(kN/m) (-)	(kN/m) (-)
*F*_A_ $\varepsilon_{P1}$	196 ± 8 0.009 ± 0.0003	913 ± 135 0.019 ± 0.002	172 ± 10 0.01± 0.001	1004 ± 41 0.016 ± 0.0005	165 ± 6 0.010 ± 0.002	1079 ± 72 0.018 ± 0.001
*F*_B_ $\varepsilon_{P2}$	47 ± 1 0.144 ± 0.002	6014 ± 180	46 ± 2 0.149 ± 0.003	6060 ± 173	46 ± 1 0.153 ± 0.002	5986 ± 209
*F*_C_	4459 ± 88	-	4338 ± 64	-	4441 ± 71	-
